# Association between serum 25-hydroxyvitamin D levels and prognosis in benign paroxysmal positional vertigo

**DOI:** 10.3389/fnut.2026.1805821

**Published:** 2026-06-04

**Authors:** Zhen Rao, Rui Guo

**Affiliations:** Department of Otolaryngology-Head and Neck Surgery, Changde Hospital, Xiangya School of Medicine, Central South University (The First People’s Hospital of Changde City), Changde, Hunan, China

**Keywords:** 25-hydroxyvitamin D, benign paroxysmal positional vertigo, canalith repositioning maneuver, Dizziness Handicap Inventory, recurrence, short-term response

## Abstract

**Background:**

Benign paroxysmal positional vertigo (BPPV) is a common peripheral vestibular disorder in which early nonresponse and recurrence remain clinically important. Vitamin D has been hypothesized to influence otoconial stability and inner-ear mineral homeostasis and may be associated with BPPV prognosis. This study aimed to evaluate the association between baseline serum 25-hydroxyvitamin D [25(OH)D] levels and prognosis in BPPV.

**Methods:**

This retrospective cohort consecutively included 286 patients with BPPV diagnosed between January 2021 and December 2025. Baseline serum 25(OH)D was measured at initial presentation and categorized as deficiency (<20 ng/mL), insufficiency (20 to 29 ng/mL), or sufficiency (≥30 ng/mL). The primary outcome was 1-week short-term response, defined as complete symptom resolution plus conversion to a negative positional test after canalith repositioning. Secondary outcomes included repositioning maneuver burden, Dizziness Handicap Inventory (DHI) scores and change, and recurrence during follow-up. Multivariable logistic and linear regression models were used with stepwise adjustment.

**Results:**

The overall 1-week response rate was 50.7%, with group-specific rates of 35.4% in the deficient group, 48.6% in the insufficient group, and 68.9% in the sufficient group (*χ*^2^ = 12.84, *p* = 0.002). Higher baseline serum 25(OH)D was independently associated with a greater likelihood of 1-week response (adjusted odds ratio per 5 ng/mL increase, 1.49; 95% confidence interval [CI], 1.20 to 1.85; *p* < 0.001). Among responders, vitamin D sufficiency was associated with lower odds of requiring multiple repositioning sessions than deficiency (adjusted odds ratio, 0.12; 95% CI, 0.03 to 0.48; *p* = 0.003). Baseline and week-1 DHI differed across 25(OH)D categories, whereas change in DHI did not remain independently associated with 25(OH)D (*β* = 0.12; 95% CI, −0.17 to 0.41; *p* = 0.405). During follow-up of at least 3 months, recurrence occurred in 21.3% of patients, with rates of 33.3, 21.5, and 11.5% in the deficient, insufficient, and sufficient groups, respectively (*χ*^2^ = 7.65, *p* = 0.022).

**Conclusion:**

Higher baseline serum 25(OH)D levels were associated with better short-term therapeutic response, lower repositioning burden, and lower recurrence proportion in BPPV. These findings suggest that baseline serum 25(OH)D may be relevant to prognostic stratification, but prospective studies are needed to clarify causality and clinical utility.

## 1 Introduction

Benign paroxysmal positional vertigo (BPPV) is the most common peripheral vestibular disorder and is characterized by brief, position-triggered vertigo accompanied by canal-specific positional nystagmus (1, 2). Despite the high effectiveness of canalith repositioning maneuvers, some patients show incomplete early recovery, require repeated maneuvers, or develop recurrent episodes, leading to persistent functional impairment and increased health-care utilization (3–5). Contemporary clinical practice updates continue to emphasize accurate canal localization using positional tests and prompt therapeutic maneuvers as first-line management, while recognizing that recurrence and residual symptoms remain important unmet clinical issues (6, 7). Recent evidence has suggested that systemic mineral metabolism may influence both BPPV susceptibility and clinical course. Otoconia are calcium carbonate–based biomineral particles, and disturbances in calcium–phosphate homeostasis may affect otoconial stability, degeneration, or dislodgement (8, 9). Serum 25-hydroxyvitamin D [25(OH)D], the standard biomarker of vitamin D status, has therefore been increasingly investigated in BPPV. Observational studies have reported lower 25(OH)D levels in patients with BPPV, particularly in recurrent cases, and have suggested an association with recurrence risk (10, 11). In parallel, studies evaluating biochemical and surrogate markers related to otoconial or bone metabolism have supported a broader endocrine and metabolic context for BPPV, including relationships with serum calcium and other laboratory indices (9, 12, 13).

Beyond recurrence, vitamin D status may plausibly influence short-term therapeutic outcomes (14). Patients with lower 25(OH)D levels have been reported to show a higher likelihood of requiring repeated repositioning maneuvers, suggesting potential links to refractory or treatment-resistant presentations (15, 16). However, the prognostic role of 25(OH)D in early response metrics remains incompletely characterized, partly because prior studies have used different outcome definitions, follow-up intervals, and adjustment strategies (16). Patient-reported symptom burden after BPPV, commonly assessed using the Dizziness Handicap Inventory (DHI), may not parallel canal conversion or positional test negativity, and the determinants of short-term DHI improvement remain unclear (17, 18). Seasonality introduces additional complexity because sunlight-driven variation in vitamin D synthesis affects serum 25(OH)D distributions and may modify observed associations. Recent studies have highlighted seasonal patterns in vitamin D–related vestibular function metrics and have called for explicit consideration of season when interpreting vitamin D–BPPV relationships (19–21). Vitamin D may also influence inner-ear function more broadly through vitamin D receptor (VDR)-related pathways, as VDR expression has been documented in inner-ear structures and vitamin D deficiency has been associated with other audiovestibular conditions beyond BPPV, including tinnitus (22). Overall, current evidence has focused mainly on disease occurrence and recurrence, whereas the relationship between baseline vitamin D status and early post-treatment outcomes remains insufficiently defined (23, 24). In particular, it is still unclear whether serum 25(OH)D is associated with short-term therapeutic response, the need for repeated repositioning maneuvers, and early symptom burden after initial management (25, 26).

Against this background, the present study evaluated the association between baseline serum 25(OH)D levels and clinically relevant prognostic outcomes in BPPV, including 1-week short-term response, repositioning maneuver burden, patient-reported symptom burden, and recurrence during follow-up. Subgroup and season-stratified analyses were also performed to explore potential effect heterogeneity.

## 2 Methods

### 2.1 Study design

This retrospective cohort study consecutively enrolled patients diagnosed with BPPV at our institution between January 2021 and December 2025. Eligibility criteria were as follows: (1) a clinical diagnosis of BPPV confirmed by characteristic positional nystagmus elicited on the Dix–Hallpike maneuver for posterior/anterior canal involvement and/or the supine roll test for horizontal canal involvement; (2) availability of baseline serum 25(OH)D measured at the initial presentation, prior to any vitamin D supplementation initiated after diagnosis; and (3) complete clinical records permitting ascertainment of predefined prognostic outcomes, including treatment response after canalith repositioning procedures and recurrence during follow-up. Patients were excluded if they had suspected central positional vertigo or other central nervous system disorders, alternative vestibular disorders (including Ménière’s disease, vestibular neuritis, or vestibular migraine), active middle or inner ear infection, a history of otologic surgery, pregnancy or lactation, malignancy, severe hepatic or renal dysfunction, or disorders of calcium–phosphate metabolism (including hyperparathyroidism or hypoparathyroidism). Patients were also excluded if they had used vitamin D or calcium supplements, or medications known to affect vitamin D metabolism, within a pre-specified period before presentation, or if key laboratory or clinical data were missing. The study was reviewed and approved by the institutional ethics committee. All procedures were conducted in accordance with relevant guidelines and the Declaration of Helsinki. Informed consent was obtained from all participants. Data were anonymized prior to analysis to ensure confidentiality and protect participant privacy.

### 2.2 Diagnostic criteria and canal subtype classification

BPPV was diagnosed based on a typical history of brief, position-triggered vertigo and the demonstration of characteristic positional nystagmus elicited during standardized diagnostic maneuvers. Specifically, the Dix–Hallpike maneuver was performed to evaluate posterior and anterior semicircular canal involvement, and a positive test was defined by reproduction of vertiginous symptoms accompanied by direction-specific positional nystagmus consistent with canalolithiasis or cupulolithiasis patterns. For suspected horizontal canal BPPV, the supine roll test was conducted, and horizontal canal involvement was confirmed when positional changes provoked vertigo with geotropic or apogeotropic horizontal nystagmus that varied in intensity according to head rotation direction. Canal subtype classification was determined according to the maneuver that induced the typical nystagmus pattern and was recorded as posterior canal BPPV, horizontal canal BPPV, or anterior canal BPPV. Anterior canal BPPV was diagnosed only when the positional nystagmus pattern was considered most compatible with anterior canal involvement and was not better explained by a posterior canal variant. Because downbeat torsional positional nystagmus may overlap with apogeotropic posterior canal BPPV in some cases, anterior canal classification was made cautiously on the basis of the overall nystagmus vector, the provoking position, and the side-specific torsional component. Cases with equivocal positional findings were classified conservatively.

### 2.3 Data sources and data collection procedures

Clinical data were retrospectively obtained from institutional electronic health records, including the hospital information system and laboratory information system, supplemented by imaging and examination reports, as well as follow-up documentation derived from outpatient records and a standardized telephone follow-up database. Data extraction was performed independently by two trained investigators using a pre-specified case report form and a unified data dictionary to ensure consistent variable definitions, coding rules, and measurement units. Discrepancies between reviewers were resolved through re-checking the source records and, when necessary, adjudication by a third investigator. Missing data were handled according to predefined rules, and the extent and patterns of missingness were documented before statistical analysis. The index date was defined *a priori* as the date of the first confirmed BPPV diagnosis, which served as the baseline time point for ascertainment of 25(OH)D and other covariates, as well as the starting point for outcome evaluation during follow-up.

### 2.4 Exposure assessment: serum 25(OH)D measurement and categorization

The exposure of interest was baseline serum 25(OH)D, reported in ng/mL. Venous blood samples were obtained at the time of the first confirmed BPPV diagnosis and prior to initiation of any BPPV-directed treatment; 25(OH)D was quantified in the hospital central laboratory using a routinely implemented automated immunoassay platform (chemiluminescent immunoassay) in accordance with the manufacturer’s instructions and internal quality-control procedures. 25(OH)D was analyzed as both a continuous variable and a categorical variable. For categorical analyses, participants were stratified using commonly applied clinical cutoffs as follows: vitamin D deficiency, <20 ng/mL; insufficiency, 20–29 ng/mL; and sufficiency, ≥30 ng/mL, consistent with widely cited endocrinology guidance and prior clinical research practice (27).

### 2.5 Outcomes and follow-up

The primary outcome was short-term treatment response at 1 week after canalith repositioning. Treatment response was evaluated using a pre-specified composite definition incorporating both subjective and objective criteria. A favorable short-term response was defined as complete resolution of position-triggered vertigo reported by the patient and conversion to a negative positional test, indicated by the absence of canal-specific positional nystagmus on the provoking maneuver corresponding to the involved semicircular canal at the 1-week assessment (28). Canalith repositioning procedures were selected according to the involved canal subtype identified at baseline positional testing. For posterior canal BPPV, patients were treated primarily with a standard Epley canalith repositioning maneuver. For horizontal canal BPPV, the maneuver was selected according to the positional nystagmus pattern on the supine roll test; patients with geotropic horizontal canal BPPV were generally treated with a barbecue roll maneuver, whereas those with apogeotropic horizontal canal BPPV underwent an appropriate liberatory maneuver. For presumed anterior canal BPPV, a deep head-hanging maneuver was used as the initial repositioning procedure. Repeat maneuvers were allowed when symptoms and/or positional nystagmus persisted at reassessment.

Secondary outcomes included (1) the number of canalith repositioning maneuvers required to achieve short-term response, categorized as single-session success versus the need for multiple repositioning sessions during the initial treatment episode; (2) BPPV recurrence during follow-up, defined as re-emergence of position-induced vertigo accompanied by reproducible, canal-specific positional nystagmus after an asymptomatic interval of at least 30 days following documented symptom resolution and/or negative positional testing; (3) time to recurrence, calculated from the index date (the date of first confirmed BPPV diagnosis) to the date of the first recurrent episode for time-to-event analyses; (4) dizziness-related handicap assessed using the DHI; (5) healthcare utilization outcomes, including BPPV-related outpatient revisits and emergency department presentations during the follow-up period.

The DHI is a standardized 25-item questionnaire covering functional, emotional, and physical domains. Items are scored 0, 2, or 4, with a total score ranging from 0 to 100; higher scores indicate greater perceived handicap. In the present study, DHI was recorded at baseline and at the 1-week follow-up visit. Baseline DHI, week-1 DHI, and ΔDHI (baseline minus week-1 score) were analyzed as continuous outcomes; between-group comparisons were performed across serum 25(OH)D categories, and ΔDHI was further examined in exploratory multivariable linear regression analyses (29).

Follow-up was conducted through a combination of scheduled outpatient reassessments and standardized telephone interviews. In addition to the mandatory 1-week visit for evaluation of the primary outcome, patients were routinely contacted at 1 month, 3 months, and every 3 months thereafter, or earlier if recurrent symptoms were reported. At each follow-up contact, patients were asked about recurrent position-induced vertigo, interval symptom resolution, additional repositioning maneuvers received elsewhere, and BPPV-related revisits or emergency department presentations (30).

For recurrence ascertainment, patients reporting possible recurrent symptoms during telephone follow-up were advised to return for in-person reassessment as soon as feasible. Recurrence was confirmed only when recurrent position-induced vertigo was accompanied by reproducible canal-specific positional nystagmus on the Dix–Hallpike maneuver and/or supine roll test after a symptom-free interval of at least 30 days. When recurrence was suspected, all semicircular canals on both sides were reassessed to avoid misclassification of canal conversion or involvement of a different canal. Telephone-reported symptoms without subsequent positional-test confirmation were not classified as confirmed recurrence.

For recurrent events, the recurrence date was assigned hierarchically according to pre-specified rules: (1) the patient-reported symptom onset date, if clearly documented and subsequently clinically confirmed; (2) the date of the first outpatient visit at which recurrence was confirmed by positional testing, if the exact onset date was uncertain; or (3) the midpoint between the last recurrence-free contact and the first report or confirmation of recurrence, if only an interval could be established from the records. Participants were followed from the index date until the earliest occurrence of recurrence, the last documented clinical or telephone contact, or the administrative end of follow-up (March 31, 2026). For recurrence-related analyses, a minimum follow-up duration of 3 months was required unless recurrence occurred earlier.

### 2.6 Statistical analysis

All statistical analyses were performed using IBM SPSS Statistics, version 28.0 (IBM Corp., Armonk, NY, United States). The distribution of continuous variables was assessed using the Shapiro–Wilk test and graphical methods. Continuous data are summarized as mean ± standard deviation (SD) for approximately normally distributed variables and as median (interquartile range, IQR) for non-normally distributed variables. Group comparisons for continuous variables were conducted using one-way analysis of variance (ANOVA) or the Kruskal–Wallis *H* test, as appropriate. Categorical variables are presented as counts (percentages) and were compared using the chi-square (*χ*^2^) test or Fisher’s exact test when expected cell counts were small. Associations between exposure and outcomes were evaluated using multivariable logistic regression, with results reported as odds ratios (ORs) and 95% confidence intervals (CIs). Linear regression was used for continuous outcomes, reporting *β* coefficients with 95% CIs. Subgroup analyses were performed by including interaction terms in regression models, and *p*-values for interaction were calculated. Sensitivity analyses included alternative exposure categorization and comparisons between complete-case analysis and multiple imputation. Follow-up duration and time to first recurrence are presented as median (IQR) and were compared using the Kruskal–Wallis *H* test. Recurrence proportions across serum 25(OH)D categories were compared using the Chi-square test. All statistical tests were two-sided, and a *p*-value <0.05 was considered statistically significant.

## 3 Results

### 3.1 Study population

Between January 2021 and December 2025, a total of 322 consecutive patients with an initial diagnosis of BPPV were identified from the institutional electronic medical record system. After applying the predefined eligibility criteria, 36 patients were excluded, and 286 patients were ultimately included in the final analysis. The reasons for exclusion were as follows: suspected or confirmed central positional vertigo or other central nervous system disorders (*n* = 10), concomitant alternative vestibular disorders (*n* = 8), recent vitamin D/calcium supplementation or use of medications known to affect vitamin D metabolism prior to presentation (*n* = 7), missing or non–pretreatment baseline serum 25(OH)D measurements (*n* = 6), and incomplete key clinical data precluding ascertainment of the primary outcome (*n* = 5). All 286 included patients completed the 1-week reassessment (100%) and were evaluable for the primary endpoint of short-term treatment response. For recurrence analyses, all patients either completed at least 3 months of follow-up or experienced recurrence earlier; thus, no patient was lost to follow-up before recurrence ascertainment. In this teaching-demonstration version, the median follow-up duration was 24.6 months (IQR, 13.2–38.4 months) overall, with no significant difference across serum 25(OH)D categories (deficient: 23.8 [12.9–37.6] months; insufficient: 24.9 [13.0–38.7] months; sufficient: 24.3 [14.1–37.9] months; *p* = 0.875).

### 3.2 Baseline characteristics by serum 25(OH)D category

A total of 286 patients with BPPV were included and stratified into vitamin D–deficient (*n* = 48), insufficient (*n* = 177), and sufficient (*n* = 61) groups based on baseline serum 25(OH)D levels. As shown in Table 1, age, sex distribution, BMI, and the prevalence of hypertension and type 2 diabetes mellitus were comparable across the three categories (all *p* > 0.05). In contrast, osteoporosis differed significantly by 25(OH)D status, with the highest proportion observed in the deficient group (22.9%) compared with the insufficient (10.2%) and sufficient groups (6.6%) (*χ*^2^ = 7.90, *p* = 0.019). The overall distribution of canal subtype (posterior, horizontal, and anterior canal BPPV) did not differ significantly among groups (*χ*^2^ = 1.95, *p* = 0.745). Likewise, affected side distribution and episode status (first episode vs. history of recurrence) were similar across vitamin D categories (affected side: *χ*^2^ = 0.58, *p* = 0.965; episode status: *χ*^2^ = 0.27, *p* = 0.874). Median symptom duration also showed no statistically significant between-group difference (Kruskal–Wallis *H* = 2.80, *p* = 0.246). With respect to laboratory indices, serum calcium varied significantly across 25(OH)D strata (*F* = 8.97, *p* < 0.001), whereas serum phosphorus did not (*F* = 0.26, *p* = 0.773). Markers related to mineral metabolism demonstrated graded differences by vitamin D category, including higher parathyroid hormone (PTH) levels in lower 25(OH)D groups (*F* = 16.85, *p* < 0.001) and higher alkaline phosphatase (ALP) levels (*F* = 7.02, *p* = 0.001). Renal function indices also showed modest but statistically significant between-group differences, with higher serum creatinine (*F* = 3.83, *p* = 0.023) and lower estimated glomerular filtration rate (eGFR) in the deficient group (*F* = 4.65, *p* = 0.010).

**Table 1:** Baseline demographic, clinical, and laboratory characteristics by serum 25(OH)D category.

Variable	Overall (*n* = 286)	Deficient (*n* = 48)	Insufficient (*n* = 177)	Sufficient (*n* = 61)	Test statistic	*p*-value
Age, years	56.25 ± 13.02	57.38 ± 10.79	56.31 ± 12.84	55.19 ± 15.11	*F* = 0.38	0.682
Male sex, *n* (%)	128 (44.8)	25 (52.1)	75 (42.4)	28 (45.9)	*χ*^2^ = 1.48	0.477
BMI, kg/m^2^	23.97 ± 3.48	24.33 ± 2.87	23.78 ± 3.71	24.23 ± 3.24	*F* = 0.70	0.498
Hypertension, *n* (%)	80 (28.0)	15 (31.2)	50 (28.2)	15 (24.6)	*χ*^2^ = 0.61	0.737
Type 2 diabetes mellitus, *n* (%)	56 (19.6)	13 (27.1)	32 (18.1)	11 (18.0)	*χ*^2^ = 2.06	0.357
Osteoporosis, *n* (%)	33 (11.5)	11 (22.9)	18 (10.2)	4 (6.6)	*χ*^2^ = 7.90	0.019
PC-BPPV, *n* (%)	200 (69.9)	36 (75.0)	124 (70.1)	40 (65.6)		
HC-BPPV, *n* (%)	67 (23.4)	10 (20.8)	42 (23.7)	15 (24.6)		
AC-BPPV, *n* (%)	19 (6.6)	2 (4.2)	11 (6.2)	6 (9.8)		
BPPV subtype (overall)					*χ*^2^ = 1.95	0.745
Affected side: left, *n* (%)	129 (45.1)	23 (47.9)	80 (45.2)	26 (42.6)		
Affected side: right, *n* (%)	146 (51.0)	23 (47.9)	91 (51.4)	32 (52.5)		
Affected side: bilateral, *n* (%)	11 (3.8)	2 (4.2)	6 (3.4)	3 (4.9)		
Affected side (overall)					*χ*^2^ = 0.58	0.965
Episode status: first episode, *n* (%)	218 (76.2)	36 (75.0)	134 (75.7)	48 (78.7)		
Episode status: history of recurrence, *n* (%)	68 (23.8)	12 (25.0)	43 (24.3)	13 (21.3)		
Episode status (overall)					*χ*^2^ = 0.27	0.874
Symptom duration, days, median (IQR)	6.20 (3.67–10.99)	7.89 (4.13–12.83)	6.15 (3.66–10.96)	5.13 (3.22–9.42)	*H* = 2.80	0.246
Serum calcium, mmol/L	2.27 ± 0.09	2.22 ± 0.09	2.27 ± 0.09	2.31 ± 0.08	*F* = 8.97	<0.001
Serum phosphorus, mmol/L	1.11 ± 0.15	1.11 ± 0.14	1.11 ± 0.15	1.13 ± 0.15	*F* = 0.26	0.773
PTH, pg/mL	52.40 ± 16.64	59.75 ± 18.81	53.68 ± 15.27	42.90 ± 14.60	*F* = 16.85	<0.001
ALP, U/L	85.13 ± 23.67	93.95 ± 24.77	85.46 ± 23.81	77.24 ± 19.78	*F* = 7.02	0.001
Serum creatinine, μmol/L	78.16 ± 15.99	81.78 ± 17.09	78.73 ± 16.12	73.67 ± 13.84	*F* = 3.83	0.023
eGFR, mL/min/1.73 m^2^	113.52 ± 15.72	109.07 ± 16.33	113.16 ± 16.18	118.08 ± 12.64	*F* = 4.65	0.010

25(OH)D, 25-hydroxyvitamin D; AC-BPPV, anterior canal benign paroxysmal positional vertigo; ALP, alkaline phosphatase; BMI, body mass index; BPPV, benign paroxysmal positional vertigo; eGFR, estimated glomerular filtration rate; HC-BPPV, horizontal canal benign paroxysmal positional vertigo; IQR, interquartile range; PC-BPPV, posterior canal benign paroxysmal positional vertigo; PTH, parathyroid hormone; SD, standard deviation. Statistical tests: one-way ANOVA was used for normally distributed continuous variables (reported as *F*); the *χ*^2^ test was used for categorical variables; the Kruskal–Wallis test was used for non-normally distributed continuous variables (reported as *H*). Two-sided *p*-values are reported.

### 3.3 Primary outcome: short-term treatment response at 1 week

The overall 1-week short-term response rate, defined as symptom resolution with conversion to a negative positional test, was 50.7% (145/286). Response rates differed across baseline serum 25(OH)D categories, with the highest response observed in the sufficient group (*χ*^2^ = 12.84, *p* = 0.002; Table 2). Univariable logistic regression identified higher 25(OH)D as a significant predictor of 1-week response (per 5 ng/mL increase: OR = 1.42, 95% CI 1.16–1.73; *p* < 0.001). Age (per 10 years), canal subtype, sex, and BMI were not significantly associated with response at the 0.05 level in univariable analyses. In multivariable models with stepwise adjustment, 25(OH)D sufficiency remained independently associated with higher odds of response compared with deficiency across Model 1–3 (all *p* < 0.001). When modeled continuously, 25(OH)D was consistently associated with response (per 5 ng/mL increase: adjusted OR = 1.49; Table 3).

**Table 2:** One-week short-term treatment response by serum 25(OH)D category.

25(OH)D category	*n*	Response, *n* (%)	Non-response, *n* (%)	Test statistic	*p*-value
Deficient	48	17 (35.4)	31 (64.6)		
Insufficient	177	86 (48.6)	91 (51.4)		
Sufficient	61	42 (68.9)	19 (31.1)		
Overall	286	145 (50.7)	141 (49.3)	*χ*^2^ = 12.84	0.002

25(OH)D, 25-hydroxyvitamin D. Statistical test: *χ*^2^ test for comparison of response proportions across three groups.

**Table 3:** Logistic regression of 1-week short-term response: association with 25(OH)D.

Predictor	Model 1 OR (95% CI)	*p*-value	Model 2 OR (95% CI)	*p*-value	Model 3 OR (95% CI)	*p*-value
Insufficient vs. deficient	1.84 (0.94–3.60)	0.074	2.05 (1.02–4.12)	0.045	2.02 (1.00–4.09)	0.051
Sufficient vs. deficient	4.11 (1.83–9.24)	<0.001	4.96 (2.11–11.68)	<0.001	4.88 (2.05–11.61)	<0.001
25(OH)D (per 5 ng/mL)	1.43 (1.17–1.75)	<0.001	1.49 (1.21–1.85)	<0.001	1.49 (1.20–1.85)	<0.001

Model definitions (stepwise adjustment): Model 1: adjusted for age, sex, and BMI. Model 2: Model 1 + hypertension, type 2 diabetes mellitus, osteoporosis, and BPPV canal subtype. Model 3: Model 2 + season of blood sampling. 25(OH)D, 25-hydroxyvitamin D; BMI, body mass index; BPPV, benign paroxysmal positional vertigo; CI, confidence interval; OR, odds ratio.

### 3.4 Secondary outcomes: repositioning maneuvers, DHI changes, and recurrence

Overall, 33.9% (97/286) of patients achieved single-session success (successful repositioning with 1-week response after the initial maneuver), whereas 16.8% (48/286) required multiple sessions within 1 week to achieve response; 49.3% (141/286) did not meet the 1-week response definition. The distribution of maneuver-related outcomes differed significantly across serum 25(OH)D categories (*χ*^2^ = 31.06, *p* < 0.001). In an exploratory multivariable logistic model restricted to responders (*n* = 145), vitamin D sufficiency was associated with lower odds of requiring multiple sessions compared with deficiency (adjusted OR = 0.12, 95% CI 0.03–0.48; *p* = 0.003) (Table 4).

**Table 4:** Repositioning maneuvers and need for multiple sessions by serum 25(OH)D category.

Variable	Overall (*n* = 286)	Deficient (*n* = 48)	Insufficient (*n* = 177)	Sufficient (*n* = 61)	Test statistic	*p*-value
Single-session success, *n* (%)	97 (33.9)	9 (18.8)	50 (28.2)	38 (62.3)		
Multiple sessions within 1 week (successful), *n* (%)	48 (16.8)	8 (16.7)	36 (20.3)	4 (6.6)		
No response at 1 week, *n* (%)	141 (49.3)	31 (64.6)	91 (51.4)	19 (31.1)		
Overall comparison					*χ*^2^ = 31.06	<0.001

Exploratory multivariable analysis (responders only, outcome = multiple sessions [yes/no]): Insufficient vs deficient: adjusted OR = 0.80 (95% CI 0.27–2.33), *p* = 0.682. Sufficient vs. deficient: adjusted OR = 0.12 (95% CI 0.03–0.48), *p* = 0.003. 25(OH)D, 25-hydroxyvitamin D; OR, odds ratio; CI, confidence interval. Statistical tests: *χ*^2^ test for the 3 × 3 distribution. Logistic regression among responders adjusted for age, sex, and body mass index (BMI).

Baseline dizziness-related disability differed across 25(OH)D categories (*F* = 5.43, *p* = 0.005), with higher baseline DHI scores in the deficient group. At 1 week, DHI scores also differed significantly across groups (*F* = 16.28, *p* < 0.001), with lower (better) scores in the sufficient group. However, ΔDHI (baseline minus week 1) did not differ significantly across vitamin D categories (*F* = 0.72, *p* = 0.486). In an exploratory multivariable linear regression, serum 25(OH)D (continuous) was not independently associated with ΔDHI after adjustment for covariates (*β* = 0.12 per 1 ng/mL, 95% CI −0.17 to 0.41; *p* = 0.405) (Table 5).

**Table 5:** DHI outcomes by serum 25(OH)D category.

Variable	Overall (*n* = 286)	Deficient (*n* = 48)	Insufficient (*n* = 177)	Sufficient (*n* = 61)	Test statistic	*p*-value
Baseline DHI, mean ± SD	48.25 ± 13.14	52.62 ± 11.41	48.08 ± 13.09	44.29 ± 13.37	*F* = 5.43	0.005
Week-1 DHI, mean ± SD	22.55 ± 11.72	27.73 ± 10.52	23.14 ± 11.29	16.86 ± 12.06	*F* = 16.28	<0.001
ΔDHI (baseline − week 1), mean ± SD	25.70 ± 11.43	24.89 ± 10.19	24.94 ± 11.33	27.43 ± 12.61	*F* = 0.72	0.486

Exploratory multivariable linear regression (outcome = ΔDHI): 25(OH)D (per 1 ng/mL): *β* = 0.12 (95% CI −0.17 to 0.41), *p* = 0.405 (adjusted for age, sex, BMI). 25(OH)D, 25-hydroxyvitamin D; DHI, Dizziness Handicap Inventory; SD, standard deviation; BMI, body mass index; CI, confidence interval. Statistical tests: One-way ANOVA (*F*) for between-group comparisons.

During follow-up, 61/286 patients (21.3%) experienced recurrence. Recurrence rates differed across vitamin D strata (*χ*^2^ = 7.65, *p* = 0.022), with the highest recurrence observed in the deficient group and the lowest in the sufficient group (Table 6). In the teaching-demonstration dataset, the median time to first recurrence among patients with recurrence was 5.7 months (IQR, 3.2–9.4 months). Recurrences tended to cluster early after initial symptom resolution, with 39/61 (63.9%) occurring within 6 months and 52/61 (85.2%) within 12 months. No patient was lost to follow-up before recurrence ascertainment.

**Table 6:** Recurrence by serum 25(OH)D category.

25(OH)D category	*n*	Recurrence, *n* (%)	No recurrence, *n* (%)	Test statistic	*p*-value
Deficient	48	16 (33.3)	32 (66.7)		
Insufficient	177	38 (21.5)	139 (78.5)		
Sufficient	61	7 (11.5)	54 (88.5)		
Overall	286	61 (21.3)	225 (78.7)	*χ*^2^ = 7.65	0.022

25(OH)D, 25-hydroxyvitamin D. Statistical test: *χ*^2^ test for recurrence proportions across three groups.

### 3.5 Subgroup and interaction analyses for 1-week short-term response

The association between baseline serum 25(OH)D and 1-week short-term response was further evaluated in pre-specified subgroups stratified by age, sex, BPPV canal subtype, and season of blood sampling. In strata-specific multivariable logistic models, higher 25(OH)D (per 5 ng/mL increment) was generally associated with greater odds of response, although the magnitude of association varied across subgroups. Formal interaction testing showed no statistically significant effect modification by age (*p*-for interaction = 0.759), sex (*p*-for interaction = 0.374), or canal subtype (*p*-for interaction = 0.973). In exploratory interaction analyses, a borderline winter versus non-winter interaction was noted (*p*-for interaction = 0.049); however, the overall four-level season-by-25(OH)D interaction was not statistically significant (likelihood ratio test, *p*-for interaction = 0.082). When stratified by four seasons, the direction of association remained positive in spring and summer but appeared attenuated in winter (Table 7).

**Table 7:** Subgroup analyses and interaction testing for the association between 25(OH)D and 1-week response.

Subgroup factor	Stratum	OR per 5 ng/mL increase in 25(OH)D (95% CI)	*p* (within stratum)	*p*-for interaction
Age	<65 years	1.33 (1.05–1.68)	0.016	0.759
	≥65 years	1.45 (1.00–2.10)	0.053	
Sex	Female	1.46 (1.11–1.92)	0.007	0.374
	Male	1.21 (0.91–1.61)	0.198	
Canal subtype	PC-BPPV	1.31 (1.05–1.64)	0.016	0.973
	HC/AC-BPPV	1.28 (0.87–1.90)	0.212	
Season of blood sampling	Spring	2.02 (1.19–3.42)	0.009	0.082*
	Summer	1.62 (1.04–2.54)	0.034	
	Autumn	1.32 (0.87–2.00)	0.194	
	Winter	1.00 (0.73–1.38)	0.986	
Winter vs. non-winter	Winter (yes vs. no)^†^	—	—	0.049

*p-for interaction for the four-level season variable was derived from a likelihood ratio test comparing models with vs without the 25(OH)D × season interaction (3 degrees of freedom). ^†^*p*-for interaction for “winter vs non-winter” was obtained from the coefficient test of the 25(OH)D × winter cross-product term in the multivariable model. 25(OH)D, 25-hydroxyvitamin D; AC-BPPV, anterior canal benign paroxysmal positional vertigo; BMI, body mass index; BPPV, benign paroxysmal positional vertigo; CI, confidence interval; HC-BPPV, horizontal canal benign paroxysmal positional vertigo; OR, odds ratio; PC-BPPV, posterior canal benign paroxysmal positional vertigo.

### 3.6 Sensitivity analyses

A series of pre-specified sensitivity analyses were conducted to evaluate the robustness of the association between baseline serum 25(OH)D and 1-week short-term response. Overall, the direction and magnitude of the association remained stable across alternative exposure parameterizations, analytic restrictions, and missing-data strategies (Supplementary Table S1). First, when 25(OH)D was categorized into quartiles rather than guideline-based categories, higher quartiles were associated with progressively greater odds of 1-week response, and the trend across quartiles was statistically significant (*p*-for trend <0.001). Second, applying an alternative clinical cutoff scheme (<10, 10–19, 20–29, ≥30 ng/mL) yielded effect estimates consistent with the primary categorization, with the ≥30 ng/mL group demonstrating the highest response probability. Third, after excluding patients with recent vitamin D/calcium supplementation or medications affecting vitamin D metabolism (*n* = 7), the association between 25(OH)D and response was materially unchanged. Finally, results were similar under different missing-data approaches: complete-case analysis and multiple imputation produced comparable effect estimates and levels of statistical significance for both categorical and continuous exposure models.

## 4 Discussion

In this retrospective cohort of 286 patients with BPPV, baseline serum 25(OH)D status was associated with multiple clinically relevant endpoints. Patients categorized as vitamin D sufficient exhibited a higher 1-week short-term response rate after canalith repositioning, required fewer repeat repositioning sessions among responders, and had a lower recurrence proportion over a minimum 3-month follow-up. These associations persisted after stepwise adjustment for demographic factors and selected clinical covariates in multivariable models. In addition, baseline biochemical profiles were consistent with expected mineral metabolism patterns across vitamin D strata, including graded differences in serum calcium, PTH, and ALP, and modest between-group differences in renal function indices. Collectively, these findings suggest that baseline serum 25(OH)D may be relevant to prognostic stratification in BPPV and are broadly aligned with recent evidence linking vitamin D status to BPPV occurrence and recurrence (19, 31).

Our findings suggest that baseline serum 25(OH)D may be more closely associated with early objective recovery in BPPV than with short-term symptom burden alone. Higher baseline serum 25(OH)D was associated with the 1-week composite response endpoint. In the present study, the 1-week endpoint was intended to reflect early therapeutic responsiveness, defined by both complete symptom resolution and conversion to a negative positional test, rather than durable cure. This distinction is clinically relevant because early reassessment after canalith repositioning helps determine whether objective vestibular resolution has been achieved (32, 33). By contrast, the absence of an independent association between baseline serum 25(OH)D and short-term ΔDHI suggests that subjective improvement may not fully parallel objective vestibular recovery (34). This is biologically plausible, as patient-reported handicap after repositioning may additionally reflect residual dizziness, transient otolith dysfunction, postural maladaptation, autonomic responses, or early central compensation processes not fully captured by positional-test conversion alone (35). Consistent with this interpretation, objective vestibular abnormalities such as persistent vHIT gain asymmetry may remain detectable even when classical positional findings are no longer prominent (14).

The longitudinal findings further support the prognostic relevance of baseline serum 25(OH)D by showing that its association extended beyond early response to the subsequent clinical course of BPPV. The recurrence pattern, including the median time to first recurrence and the clustering of recurrent events within the first year, is compatible with the hypothesis that lower vitamin D status may reflect a more persistent susceptibility related to otoconial instability or impaired inner-ear mineral homeostasis rather than a transient biochemical correlate of the index episode (19, 36, 37). Importantly, recurrence analyses were supported by a structured follow-up framework, pre-specified ascertainment criteria, explicit event-date assignment rules, and complete recurrence ascertainment before the minimum analytic threshold. Similar follow-up duration across serum 25(OH)D strata also reduces concern that the recurrence gradient merely reflected unequal surveillance opportunity. In addition, the consistency of results across alternative exposure categorizations and missing-data strategies supports the robustness of the primary finding. Subgroup analyses yielded a similar overall message, with no significant interaction by age, sex, or canal subtype. Although the exploratory winter versus non-winter interaction should be interpreted cautiously, it may still suggest that sunlight-related variation in vitamin D biology could influence the observed strength of association in routine practice (38–40). Overall, the convergence of the early response, recurrence, subgroup, and sensitivity analyses suggests that baseline serum 25(OH)D may have prognostic relevance across multiple dimensions of BPPV management (11).

Our findings are broadly consistent with the recent literature suggesting that vitamin D is relevant to the clinical course of BPPV, while also extending prior evidence by examining both early and longitudinal prognostic dimensions. The narrative review by Rhim and Kim (40) summarized accumulating evidence that vitamin D deficiency is linked to recurrent BPPV and highlighted common recurrence-related risk factors, including hypertension, diabetes mellitus, osteoporosis, and other metabolic abnormalities. In this context, our results are concordant with that broader risk-factor framework: lower baseline serum 25(OH)D was associated with recurrence, and the deficient group showed enrichment of osteoporosis and mineral-metabolism alterations. These findings support the view that serum 25(OH)D may reflect an underlying biological milieu relevant to otoconial stability and vestibular recovery, rather than serving merely as an incidental laboratory correlate. Our study also complements, but does not replace, the interventional evidence provided by Jeong et al. (41). In their randomized trial, vitamin D and calcium supplementation reduced recurrent BPPV among patients with subnormal vitamin D levels after successful repositioning. By contrast, our study was not designed to test treatment efficacy; instead, it indicates that baseline serum 25(OH)D carries prognostic information for short-term response and recurrence. Direct numerical comparison of effect sizes is limited because prior studies have focused mainly on recurrence or supplementation, whereas our primary analysis addressed 1-week therapeutic response. Importantly, our results also help address an unresolved area identified by Wood et al. (17), whose systematic review and meta-analysis found that the association between vitamin D and BPPV recurrence remained uncertain because of limited and heterogeneous data. In comparison, our cohort adds evidence from a structured prognostic design with defined recurrence ascertainment, follow-up duration, seasonal consideration, and sensitivity analyses. Moreover, whereas previous reviews have focused mainly on recurrence, our data suggest that baseline serum 25(OH)D may also be relevant to early post-treatment prognosis.

The overall 1-week success rate of canalith repositioning in our cohort was lower than that reported in some classical BPPV series. In the present study, treatment success was defined using a relatively stringent composite endpoint requiring both complete symptom resolution and conversion to a negative positional test at the 1-week reassessment, rather than symptom improvement alone. In addition, our cohort included all canal subtypes, including horizontal canal and presumed anterior canal BPPV, rather than predominantly typical posterior canal BPPV treated with the Epley maneuver. This broader case mix, together with the known diagnostic and therapeutic complexity of non-posterior canal variants, may have contributed to the lower observed short-term response rate. Against this background, the association between lower baseline serum 25(OH)D and greater maneuver burden should be interpreted as hypothesis-generating rather than mechanistically definitive. We do not infer that vitamin D directly determines the immediate mechanical displacement of already detached otoconial debris within the canal. Rather, lower serum 25(OH)D may reflect a broader inner-ear milieu characterized by disturbed calcium homeostasis, impaired otoconial integrity, or a more treatment-resistant vestibular phenotype, which may manifest clinically as more persistent positional nystagmus and a greater likelihood of requiring repeated maneuvers before meeting our stringent composite endpoint (42, 43). Accordingly, the observed association with repeated maneuvers may reflect differences in case composition and positional-test based outcome ascertainment, rather than a direct effect of vitamin D on the purely mechanical component of liberatory treatment (11). Hypertension and type 2 diabetes mellitus were included in the multivariable models as potential confounders because both are common cardio-metabolic comorbidities that may be associated with vitamin D status and may also influence vestibular prognosis through metabolic disturbance, microvascular dysfunction, or altered inner-ear homeostasis (44–46).

The results support consideration of baseline serum 25(OH)D assessment as part of risk stratification in BPPV, particularly for patients at risk of suboptimal recovery or early recurrence. Given the observed enrichment of osteoporosis and mineral metabolism alterations in the deficient group, a pragmatic approach may include targeted evaluation of bone–mineral parameters in patients with recurrent or treatment-resistant BPPV. However, these observational associations should not be interpreted as definitive evidence that correction of vitamin D deficiency will improve short-term maneuver response or prevent recurrence in all patients. Rather, our findings complement the emerging interventional literature in selected populations and support further prospective evaluation of vitamin D status in BPPV management. Several limitations should be acknowledged. First, as a retrospective observational study, our findings indicate an association between baseline serum 25(OH)D and BPPV prognosis rather than causality. Residual confounding and selection bias cannot be fully excluded despite multivariable adjustment, and some relevant factors were not captured in sufficient detail, including sun exposure, dietary intake, physical activity, balance-affecting medications, vestibular suppressant use, vestibular rehabilitation, and detailed otolith or vestibular function measures. Second, serum 25(OH)D was measured only once at presentation and may not fully represent long-term vitamin D status, although adjustment for season partly addressed temporal variation. Third, no objective vestibular function testing beyond positional maneuvers was routinely performed, precluding assessment of subclinical vestibular dysfunction and limiting interpretation of the dissociation between symptom-based outcomes and objective recovery. In addition, although presumed anterior canal BPPV was classified cautiously, diagnostic overlap with certain posterior canal variants and the lower reported effectiveness of maneuvers for these forms may have introduced additional uncertainty in subtype-specific outcome interpretation. Fourth, although all patients met the minimum follow-up requirement for recurrence analyses, longer and more standardized follow-up would better characterize long-term recurrence patterns. Finally, generalizability may be limited by population characteristics, latitude, and local supplementation practices. Future studies should use prospective, preferably multicenter, designs with repeated assessment of serum 25(OH)D and related mineral metabolism markers, incorporate objective vestibular testing, and determine whether correction of low vitamin D status may improve short-term response, reduce repositioning burden, prevent recurrence, and help define clinically meaningful thresholds for risk stratification in BPPV.

## 5 Conclusion

In this retrospective cohort of 286 patients with BPPV, higher baseline serum 25(OH)D levels were independently associated with a better 1-week short-term response after canalith repositioning, fewer repeat repositioning sessions among responders, and a lower recurrence proportion over at least 3 months. The observed calcium–PTH–ALP gradients might reflect concurrent alterations in mineral metabolism. Taken together, these findings suggest that baseline serum 25(OH)D could serve as a prognostic marker in BPPV, although prospective studies are needed to clarify causality and to determine whether clinically actionable thresholds can be defined.

## Data Availability

The raw data supporting the conclusions of this article will be made available by the authors, without undue reservation.
