# Supplementary material for: Association between serum 25-hydroxyvitamin D levels and prognosis in benign paroxysmal positional vertigo
Source: Front Nutr. 2026 Jun 4;13:1805821. doi: 10.3389/fnut.2026.1805821 (PMC13275238; doi:10.3389/fnut.2026.1805821)
Supplement: Supplementary file 1 [file Table_1.DOCX]

Supplementary Table S1. Sensitivity analyses for the association between baseline serum 25(OH)D and 1-week short-term response.

| Sensitivity scenario | Sample size (n) | Exposure parameterization | Effect estimate (95% CI) | P value | Notes |
| --- | --- | --- | --- | --- | --- |
| Primary analysis (reference) | 286 | Per 5 ng/mL increase | OR 1.49 (1.20–1.85) | <0.001 | Model 3 adjustment set |
|  | 286 | Categories (ref: deficient) | Sufficient vs deficient: OR 4.88 (2.05–11.61) | <0.001 | Model 3 |
| Alternative categorization: quartiles | 286 | Q4 vs Q1 | OR 3.62 (1.82–7.20) | <0.001 | Model 3 |
|  | 286 | Q3 vs Q1 | OR 2.06 (1.05–4.04) | 0.036 | Model 3 |
|  | 286 | Q2 vs Q1 | OR 1.32 (0.68–2.58) | 0.413 | Model 3 |
|  | 286 | Trend across quartiles | OR per quartile 1.46 (1.18–1.81) | 0.001 | Ordinal term |
| Alternative clinical cutoffs | 286 | ≥30 vs <10 ng/mL | OR 5.21 (1.63–16.67) | 0.005 | Model 3 |
|  | 286 | 20–29 vs <10 ng/mL | OR 2.51 (0.82–7.72) | 0.108 | Model 3 |
|  | 286 | 10–19 vs <10 ng/mL | OR 1.38 (0.44–4.33) | 0.579 | Model 3 |
| Exclude recent supplements/medications | 279 | Per 5 ng/mL increase | OR 1.52 (1.21–1.91) | <0.001 | Model 3; excluded n=7 |
|  | 279 | Sufficient vs deficient | OR 4.94 (2.02–12.12) | <0.001 | Model 3 |
| Missing-data strategy: complete-case | 270 | Per 5 ng/mL increase | OR 1.47 (1.17–1.85) | 0.001 | Complete-case set |
| Missing-data strategy: multiple imputation | 286 | Per 5 ng/mL increase | OR 1.48 (1.19–1.84) | <0.001 | 20 imputations |
|  | 286 | Sufficient vs deficient | OR 4.79 (2.02–11.33) | <0.001 | Imputed covariates |

Abbreviations: 25(OH)D, 25-hydroxyvitamin D; CI, confidence interval; OR, odds ratio; Q, quartile.

Statistical notes: All odds ratios were derived from multivariable logistic regression with 1-week response as the dependent variable. “Model 3” corresponds to stepwise adjustment for age, sex, BMI, hypertension, type 2 diabetes mellitus, osteoporosis, BPPV canal subtype, and season of blood sampling. Multiple imputation was performed using chained equations with 20 imputations, pooling estimates using Rubin’s rules.
